# Boxwood phyllosphere fungal and bacterial communities and their differential responses to film-forming anti-desiccants

**DOI:** 10.1186/s12866-023-02956-0

**Published:** 2023-08-12

**Authors:** Xiaoping Li, Olanike Omolehin, Ginger Hemmings, Hsien Tzer Tseng, Amanda Taylor, Chad Taylor, Ping Kong, Margery Daughtrey, Douglas Luster, Fred Gouker, Chuanxue Hong

**Affiliations:** 1 Hampton Roads Agricultural Research and Extension Center, Virginia Tech, Virginia Beach, VA USA; 2 Plant Industry Division, North Carolina Department of Agriculture and Consumer Services, Dobson, NC USA; 3 Plant Industry Division, North Carolina Department of Agriculture and Consumer Services, Raleigh, NC USA; 4 North Carolina University Cooperative Extension, Morganton, NC USA; 5 Plant Industry Division, North Carolina Department of Agriculture and Consumer Services, Boone, NC USA; 6 Long Island Horticultural Research and Extension Center, Cornell University, Riverhead, NY USA; 7 Foreign Disease-Weed Science Research Unit, USDA-ARS-NEA, Fort Detrick, MD USA; 8 U.S. National Arboretum, Floral and Nursery Plants Research Unit, USDA-ARS, Beltsville, MD USA

**Keywords:** Anti-transpirant, Di-1-p-menthene, Phytobiomes, Boxwood, Crop protection, Systems approach, Nanopore MinION sequencing

## Abstract

**Background:**

Anti-desiccant is a class of agrochemicals widely used to protect plants from water stresses, rapid temperature variations, heat and sunburn, frost and freeze damages, transplant shock, and pathogen and pest attack. Although anti-desiccants are generally considered non-toxic to organisms, it is unclear whether they may impact the phyllosphere microbial communities. In this study, three film-forming anti-desiccant products, TransFilm, Vapor Gard, and Wilt-Pruf were applied to the canopy of two boxwood cultivars ‘Vardar Valley’ and ‘Justin Brouwers’ on April 13 and August 26, 2021. Shoot samples were collected from boxwood plants treated with each of the three products, as well as nontreated control on June 16, August 26 (before the second treatment), and October 18. Microbial and plant genomic DNA was isolated together and 16S rRNA gene and the extended internal transcribed spacer regions were amplified with PCR and sequenced on a Nanopore MinION platform for bacterial and fungal identification.

**Results:**

Bacterial communities were more diverse than fungal communities. At the phylum level, the boxwood phyllosphere was dominated by *Proteobacteria* and *Ascomycota*; at the genus level, *Methylobacterium* and *Shiraia* were the most abundant bacteria and fungi, respectively. Among the three film-forming anti-desiccants, Vapor Gard and Wilt-Pruf had more impact than TransFilm on the microbial communities. Specifically, broader impacts were observed on fungal than bacterial community composition and structure, with most affected fungi being suppressed while bacteria promoted.

**Conclusion:**

This study addressed several major knowledge gaps regarding boxwood phyllosphere microbiota and the impact of anti-desiccants on plant microbiome. We identified diverse microbial communities of boxwood, a major evergreen woody crop and an iconic landscape plant. We also found differential effects of three film-forming anti-desiccants on the composition and structure of bacterial and fungal communities. These findings advanced our understanding of the associated microbiome of this landmark plant, enabling growers to fully utilize the potentials of microbiome and three anti-desiccants in improving boxwood health and productivity.

**Supplementary Information:**

The online version contains supplementary material available at 10.1186/s12866-023-02956-0.

## Background

Plant phyllosphere is colonized by numerous bacteria and fungi, with some performing important functions linked to plant health and productivity. These functions range from mitigating environmental stresses to suppressing plant pathogens, including antagonizing plant pathogens [1], inducing plant systemic resistance [2], improving nutrient acquisition [3, 4], and synthesizing plant growth hormones [5]. Knowing how biotic and abiotic factors may impact phyllosphere microbial communities is fundamental for leveraging beneficial microbes to crop health and productivity.

Boxwood is a widely cultivated ornamental evergreen crop with societal and economic importance [6, 7], and the understanding of its phyllosphere microbial communities has recently improved. Kong et al. [8] showed that the foliar culturable bacterial and fungal endophytes of English boxwood (*Buxus sempervirens* ‘Suffruticosa’) were associated with the cultivar’s differential tolerance to the boxwood blight disease. They also isolated a *Burkholderia* SSG strain from boxwood leaf tissues and showed its beneficial activities in nutrient acquisition [3] and pathogen antagonism [9]. Recently, we revealed diverse bacterial communities on the boxwood shoot surface and in the internal tissue and found that canopy cover sprayed with contact and systemic fungicides had a broad and strong impact on the epiphytic bacterial communities [10]. These studies have advanced our knowledge of the biology of boxwood and also highlighted the importance of evaluating other agrochemicals for their potential impacts on the boxwood phyllosphere microbiota—an essential step to establishing a systems approach to utilize the full potential of microbiomes for better boxwood management.

Anti-desiccants (also known as anti-transpirants) are a class of agrochemicals broadly used in agriculture and horticulture to protect plants from drought stress [11, 12], sun burn [13, 14], transplant shock [15, 16], winter injury [17, 18], and plant diseases [19–21]. As classified according to their modes of action, three types of anti-desiccants are available on the market: (1) film-forming; (2) reflective; and (3) metabolic [22]. Among them, film-forming anti-desiccant is the most commonly applied. The mechanism of film-forming anti-desiccants is to establish a thin polymeric barrier on the leaf or plant surface to reduce plant water loss during transpiration [22]. A common active ingredient of the film-forming anti-desiccants is di-1-p-menthene (also known as pinolene), a terpenic polymer derived from conifer resin and can be emulsified with water [23]. After spraying, it generates a glossy, flexible, and transparent coating that is impermeable to water vapors [24] and also reduces gas exchange [25], resulting in a decreased net photosynthesis [26]. However, film-forming anti-desiccants are generally considered to be non-toxic to plants [27] and other organisms [28], and can persist in effectiveness for months [29].

Unlike pesticides, the impact of anti-desiccants on plant microbiomes remains unknown. While most studies have focused on the changes in plant physiochemistry [11, 30, 31], growth [26], and yields [25] as affected by anti-desiccants, several others have explored the role that film-forming polymers may play in disease management. Elad et al. [32] showed that di-p-methene containing products Vapor Gard and Wilt-Pruf reduced the incidence of powdery mildew up to 82% and 55% in pot grown cucumber. Similarly, Haggag [19] demonstrated suppressing effects of several film-forming anti-desiccants on cucumber downy mildew in a greenhouse study. Most importantly, he provided electron microscopic evidence to show that kaolin anti-desiccant inhibited spore germination and disrupted the sporangia formation of the casual pathogen *Pseudoperonospora cubensis* [19]. The controlling effect of anti-desiccants on many other foliar diseases was also reported for various plants [20, 33–35]. These studies together imply that the film-forming anti-desiccants may direct or indirectly act against fungi with unclear mechanisms. However, it is still unknown whether and to what degree film-forming anti-desiccants may impact plant microbial communities, particularly those colonizing the phyllosphere. Answering these questions is important for growers to properly use this class of agrochemicals for crop health and productivity while maximizing the benefits of the plant microbiomes.

Film-forming anti-desiccants are frequently applied to care for boxwood overwintering, summer drought, and transplant shocks in landscape and nurseries. The objectives of this study were to characterize the phyllosphere microbial communities and investigate how film-forming anti-desiccants may impact the microbiota. Three anti-desiccant products were applied to the canopy of two boxwood cultivars ‘Vardar Valley’ and ‘Justin Brouwers’, and boxwood shoots were taken two and four months after treatments. Microbial and plant genomic DNA was extracted, and the full length 16 S ribosomal RNA (rRNA) gene and the extended internal transcribed spacer (ITS) regions were amplified, and resultant amplicons were sequenced on a Nanopore MinION® platform for bacterial and fungal identification.

## Results

### Nanopore sequencing summary

Nanopore MinION sequencing generated a total of 25,391,330 raw reads, with 13,942,987 and 11,448,343 reads for 16 S rRNA and ITS amplicons, respectively. After removing boxwood chloroplast sequences and filtering out samples less than 1,000 reads and operational taxonomic units (OTUs) less than 10 reads, 67,693 and 5,667,266 sequences were retained for 16 S rRNA and ITS amplicons, respectively. Average sequencing coverage was 90.0% for 16 S rRNA (n = 96) and 99.9% for ITS amplicons (n = 96). Rarefaction curves based on the clean reads suggested sequencing for ITS reached near-plateau but the depth of 16 S rRNA was comparably shallow due to the removal of 13,298,594 chloroplast sequences (Fig. S1).

### Taxonomic compositions of phyllosphere microbial communities

For the total of 96 samples, the 16 S rRNA reads were assigned to 327 genera, 182 families, 105 orders, 49 classes, and 24 phyla for bacterial communities; the ITS reads were assigned to 328 genera, 198 families, 89 orders, 27 classes, and 2 phyla for fungal communities.

**Fig. 1:**
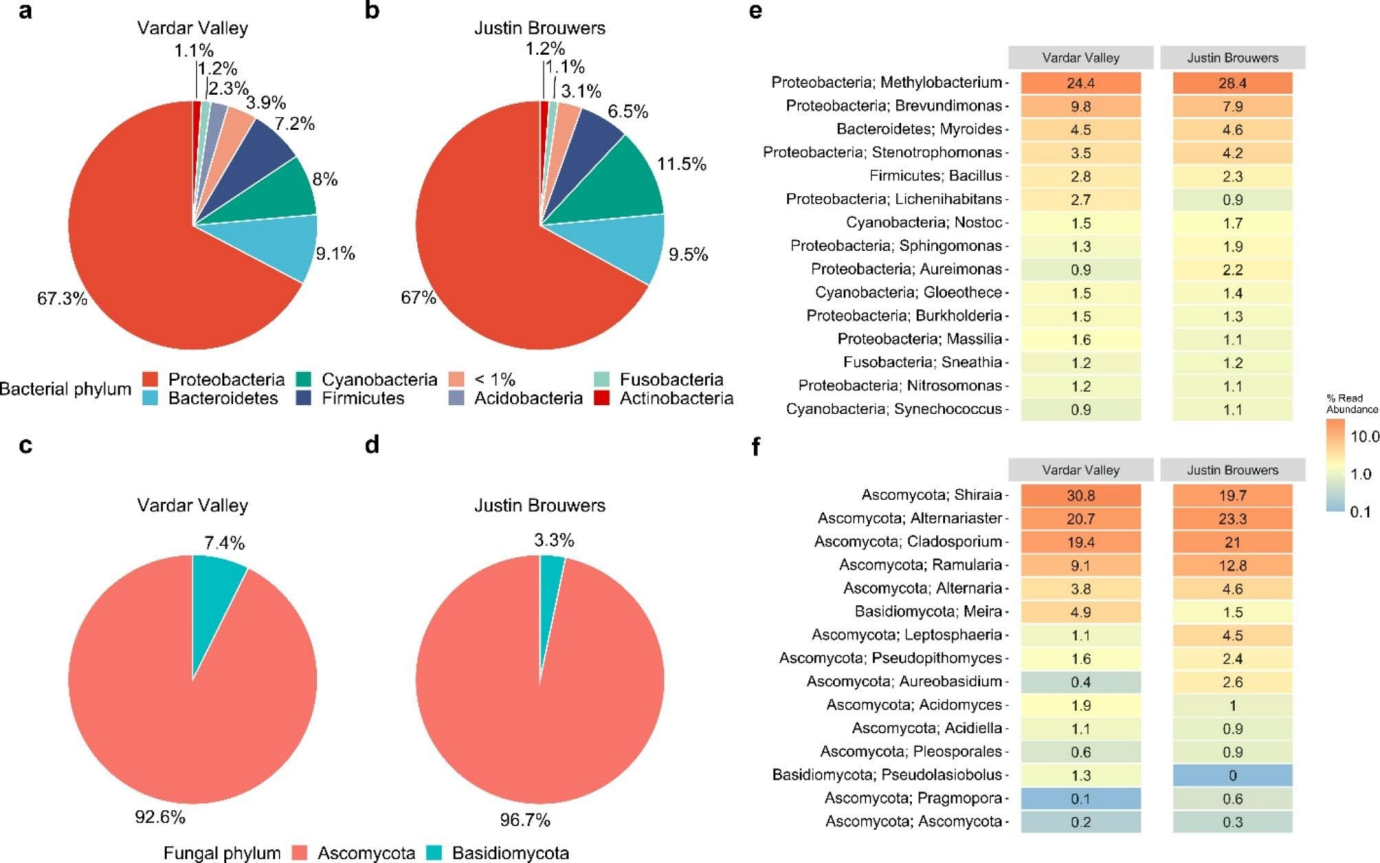
Pie charts showing bacterial (**a** and **b**) and fungal (**c** and **d**) phylum compositions in ‘Vardar Valley’ and ‘Justin Brouwers’ boxwood shoots. Heatmap showing the relative abundance of the top 15 predominant bacterial (**e**) and fungal genera (**f**) in the boxwood cultivars

Bacterial communities were diverse with slight variation in relative abundance between the two cultivars. At the phylum level, *Proteobacteria* dominated the phyllosphere bacterial communities of both cultivars with relative abundance over 65%, followed by *Bacteroidetes*, *Cyanobacteria*, *Firmicutes*, *Fusobacteria*, and *Actinobacteria* (Fig. 1a and b). Between the two cultivars, the relative abundance of *Bacteroidetes*, *Cyanobacteria*, and *Actinobacteria* were 3.5–0.1% higher in cultivar ‘Justin Brouwers’ while *Firmicutes* and *Fusobacteria* were 0.7% and 0.1% higher in ‘Vardar Valley’. Notably, the relative abundance of *Acidobacteria* was 2.3% in ‘Vardar Valley’ but less than 1.0% in ‘Justin Brouwers’. At the genus level, *Methylobacterium*, *Brevundimonas*, *Myroides*, *Stenotrophomonas*, *Bacillus*, *Lichenihabitans*, *Nostoc*, *Sphingomonas*, *Aureimonas*, *Gloeothece*, *Burkholderia*, *Massilia*, *Nitrosomonas*, and *Synechococcus* were the predominant genera. Of these genera, the relative abundance of *Brevundimonas*, *Bacillus*, *Lichenihabitans*, and *Massilia* were 0.5–1.9% higher in the ‘Vardar Valley’ than ‘Justin Brouwers’ (Fig. 1e). In contrast, the relative abundance of *Methylobacterium*, *Stenotrophomonas*, *Sphingomonas*, and *Aureimonas* were 0.6–4.0% higher in the ‘Justin Brouwers’ than ‘Vardar Valley’.

Fungal communities were less diverse than the bacterial communities and dominated by only two phyla: *Ascomycota* and *Basidiomycota*. Particularly, *Ascomycota* dominated the boxwood phyllosphere with relative abundance over 92.0% in both cultivars (Fig. 1c and d). On the other hand, the relative abundance of *Basidiomycota* was 7.4% in ‘Vardar Valley’, about 4.0% higher than that of ‘Justin Brouwers’. At the genus level, *Shiraia*, *Alternariaster*, *Cladosporium*, *Ramularia*, *Alternaria*, *Meira*, *Leptosphaeria*, *Pseudopithomyces*, *Aureobasidium*, *Acidomyces*, *Acidiella*, *Pleosporales*, *Pseudolasiobolus*, *Pragmopora*, and an unknown genus from phylum *Ascomycota* were the predominant fungi (Fig. 1f). Of these genera, the relative abundance of *Shiraia*, *Meira*, *Acidomyces*, and *Pseudolasiobolus* were 0.9–11.1% greater in ‘Vardar Valley’ than in ‘Justin Brouwers’, while the relative abundance of *Alternariaster*, *Cladosporium*, *Ramularia*, *Alternaria*, *Leptosphaeria*, *Pseudopithomyces*, *Aureobasidium*, and *Pragmopora* were 0.5–3.7% higher in ‘Justin Brouwers’ than in ‘Vardar Valley’.

The bacterial and fungal genera detected were rather consistent across three sampling times, although the total number of genera seen at each time differed slightly. For example, 296, 280, and 301 bacterial genera were identified from ‘Vardar Valley’ boxwood collected in June, August, and October, respectively, with 250 genera consistently observed across all three months (Fig. S2a). Likewise, 299, 302, and 299 bacterial genera were detected from ‘Justin Brouwers’ boxwood in June, August, and October, respectively, with 259 being seen consistently across all three months (Fig. S2b). For fungal communities, 274, 286, and 312 genera were identified from ‘Vardar Valley’ boxwood in June, August, and October, respectively, with 243 genera consistently present across all three months (Fig. S2c). Similarly, 273, 269, and 280 genera were identified from ‘Justin Brouwers’ boxwood in June, August, and October, respectively, with 222 genera present in all three months (Fig. S2d).

### Community compositional changes in response to anti-desiccant treatments

Overall, anti-desiccants had more pronounced effects on the fungal than bacterial communities. Analysis of Compositional of Microbiomes with Bias Correction (ANCOM-BC) identified 96 fungal and 62 bacterial genera differentially abundant when comparing each anti-desiccant to the nontreated control across the two cultivars and the three sampling seasons.

**Fig. 2:**
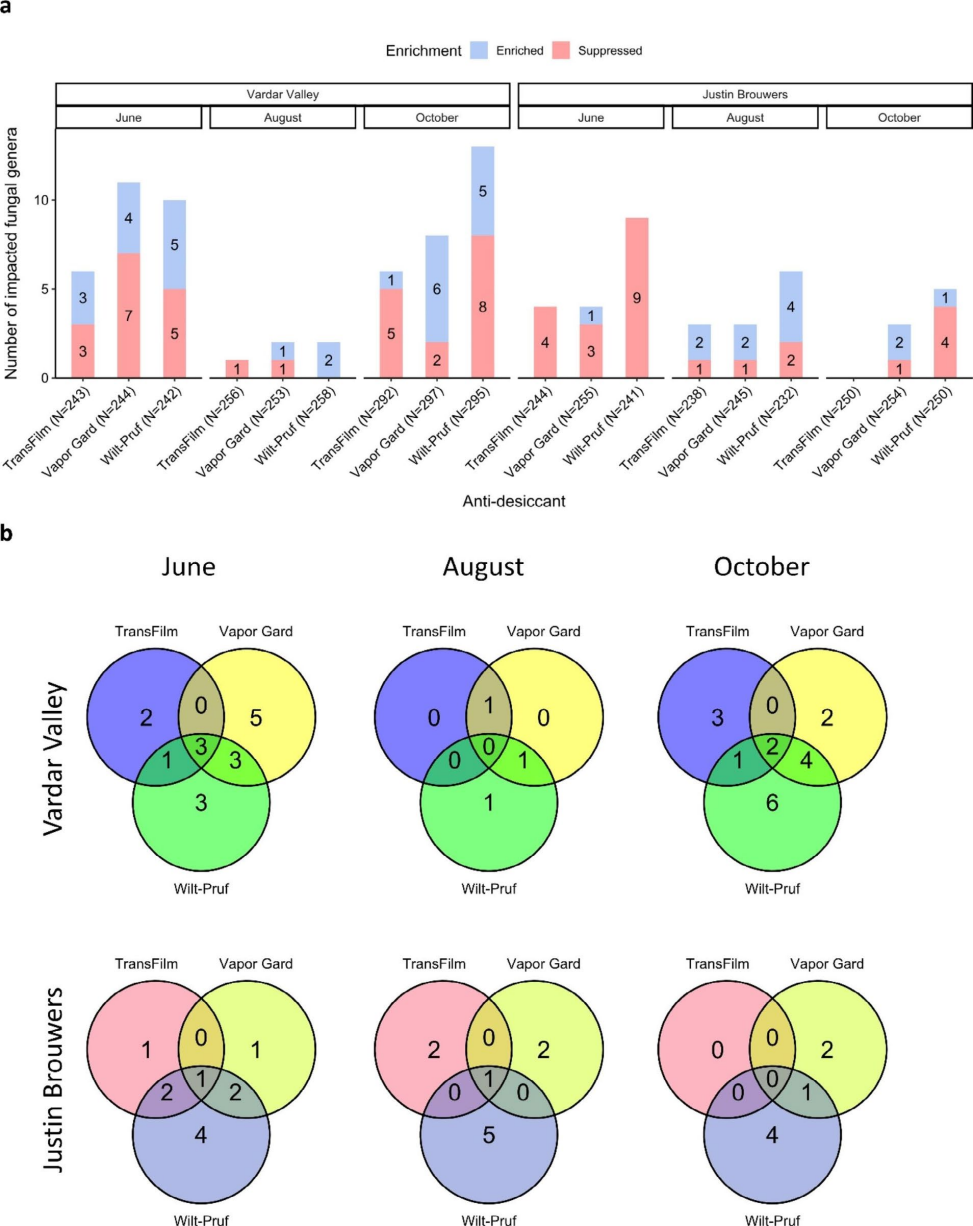
(**a**) Barplot showing the number of significantly (Adjusted *P* value < 0.05) enriched and suppressed fungal genera in boxwood shoots by anti-desiccant treatment when compared to the nontreated control by cultivar and sampling month. The number was based on the sum of all replicates and the total number of genera is indicated in the parenthesis for each comparison. (**b**) Venn diagram showing the number of differentially abundant fungal genera in boxwood shoots unique to or shared among anti-desiccants by cultivar and sampling month

The effect of anti-desiccant was largely suppressive on fungal communities. ANCOM-BC identified 59 and 37 fungal genera with differential abundance for ‘Vardar Valley’ and ‘Justin Brouwers’, respectively, with 54.2% and 67.6% of them were suppressed with anti-desiccants compared to the nontreated controls (Fig. 2a).

**Fig. 3:**
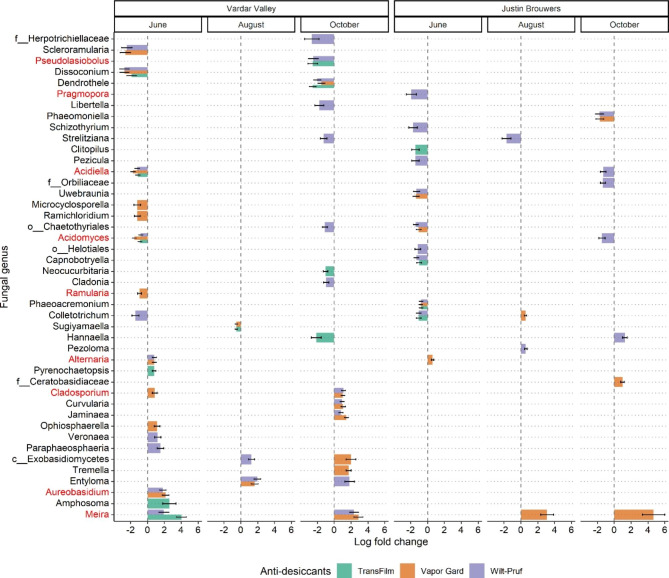
Barplot showing the significant (Adjusted *P* value < 0.05) fungal genera with log fold abundance change ≥ 0.5 or ≤ -0.5. Taxa that are written in red are the predominant genera

Vapor Gard and Wilt-Pruf had broader and stronger impact on fungal communities than TransFilm. Specifically, Vapor Gard and Wilt-Pruf each affected more fungal genera than TransFilm in both cultivars (Fig. 2b). For example, Vapor Gard and Wilt-Pruf impacted 20 and 24 genera, respectively, while TransFilm only affected 11 genera in ‘Vardar Valley’ boxwood (Fig. 3). Likewise, Vapor Gard and Wilt-Pruf impacted 9 and 16 genera, respectively, while TransFilm only affected 4 genera in ‘Justin Brouwers’ boxwood (Fig. 3). These impacted fungi included several predominant genera: *Pseudolasiobolus*, *Pragmopora*, *Acidiella*, *Acidomyces*, *Ramularia*, *Alternaria*, *Cladosporium*, *Aureobasidium*, and *Meira* (Fig. 3). Among them, *Pseudolasiobolus*, *Pragmopora*, *Acidiella*, *Acidomyces*, and *Ramularia* appeared to be negatively impacted by anti-desiccant, while *Alternaria*, *Cladosporium*, *Aureobasidium*, and *Meira* were positively affected by anti-desiccant.

Fungal communities were more responsive to anti-desiccant treatments two months after application than four months later. For both cultivars, ANCOM-BC identified 28 and 25 differentially abundant fungal genera in June and October, two months post-treatment, while 13 genera differed in August, four months after the first treatment in April (Fig. 2b). Additionally, 26 and 21 of the affected fungal genera in June and October had a log fold change of abundance ≥ 0.5 or ≤ -0.5, while it was only 7 genera in August (Fig. 3).

**Fig. 4:**
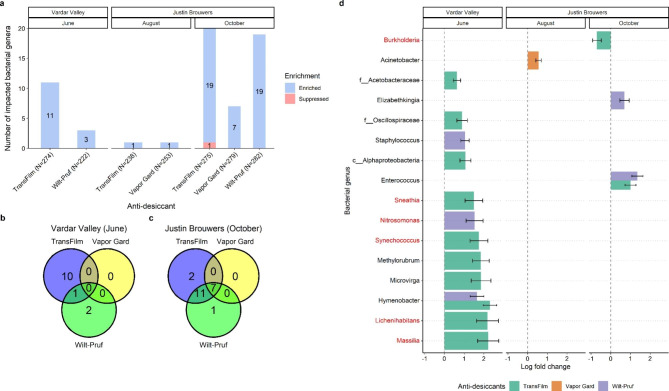
(**a**) Barplot showing the number of significantly (Adjusted *P* value < 0.05) enriched and suppressed bacterial genera in boxwood shoots by anti-desiccant treatment when compared to the nontreated control. The number was based on the sum of all replicates and the total number of genera is indicated in the parenthesis for each comparison. (**b** and **c**) Venn diagram showing the number of differentially abundant bacterial genera unique to or shared among anti-desiccants for the cultivar ‘Vardar Valley’ in June and ‘Justin Brouwers’ in October. (**d**) Barplot showing the significant bacteria genera with log fold abundance change ≥ 0.5 or ≤ -0.5. The predominant genera are written in red. Only sampling months or anti-desiccant treatments with significant impact were included

Unlike fungal communities, bacterial communities were mostly promoted by the selected anti-desiccants. Specifically, TransFilm and Wilt-Pruf promoted 14 bacterial genera in cultivar ‘Vardar Valley’ in June (Fig. 4a). Of these promoted genera, ten were solely promoted by TransFilm and two by Wilt-Pruf, while only one genus was promoted by both TransFilm and Wilt-Pruf (Fig. 4b). These promoted genera included several predominant bacteria: *Massilia*, *Lichenihabitans*, *Synechococcus*, *Nitrosomonas*, and *Sneathia* (Fig. 4d). Similarly, TransFilm, Vapor Gard, and Wilt-Pruf respectively enriched 19, 7, and 19 genera in cultivar ‘Justin Brouwers’ in October, with TransFilm suppressing only one genus. Of the impacted genera, seven were impacted by all three anti-desiccants and 11 were affected by TransFilm and Wilt-Pruf. Only two were solely impacted by TransFilm and one by Wilt-Pruf (Fig. 4c). The affected bacterial genera with a log fold change of abundance ≥ 0.5 or ≤ -0.5 were *Enterococcus*, *Elizabethkingia*, and *Burkholderia* (Fig. 4d); and *Burkholderia*, the only predominant bacterial genus, was suppressed by TransFilm. Notably, none of the genera were affected by the three anti-desiccants in August and October in ‘Vardar Valley’ and in June in ‘Justin Brouwers’.

### Changes in community structure responding to anti-desiccant treatments

**Fig. 5:**
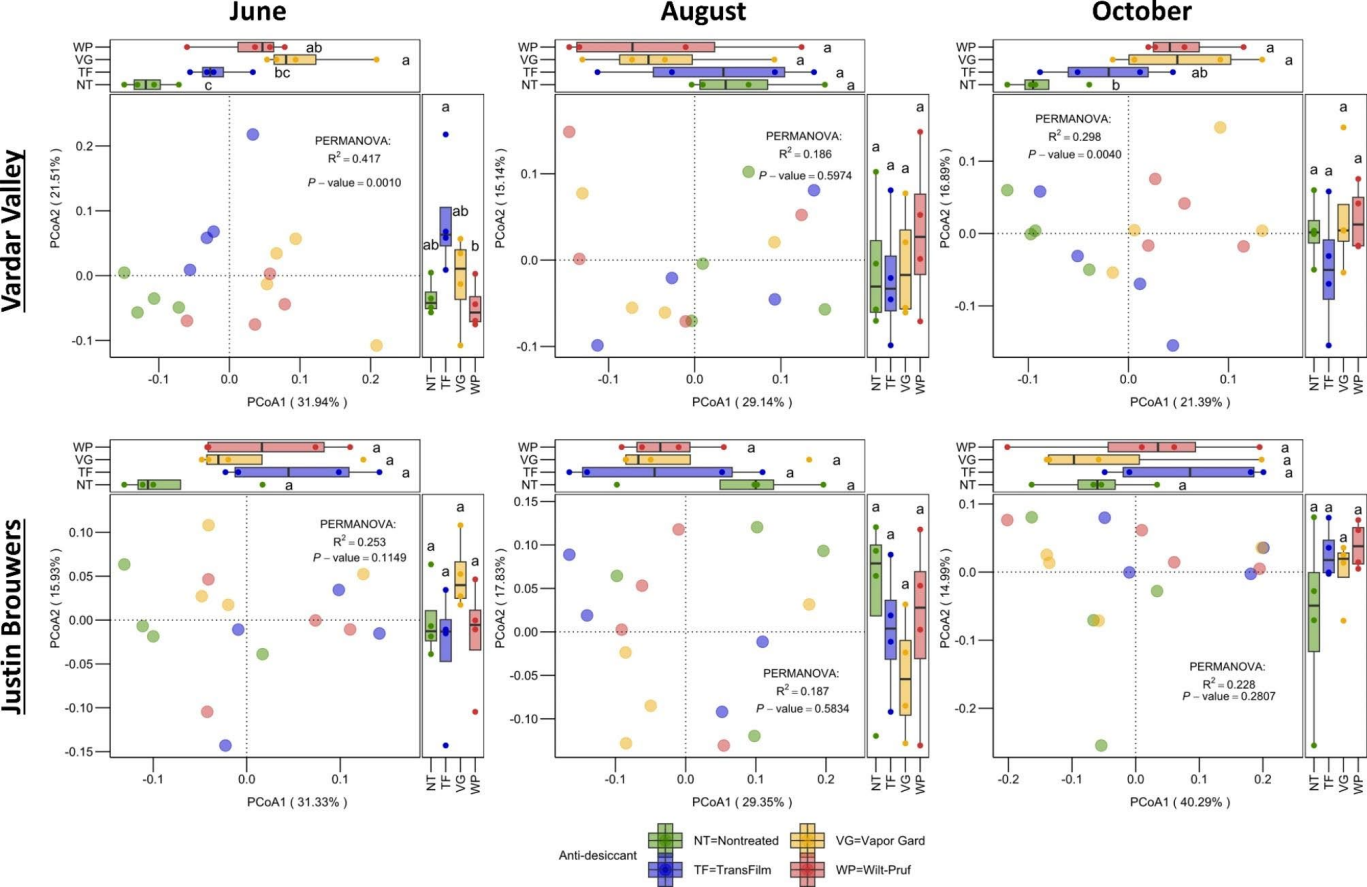
Fungal community structure of two boxwood cultivars as affected by anti-desiccant treatments in the three sampling months. Anti-desiccant effect as analyzed by PERMANOVA is shown in the figure. Boxplot on x- and y- side demonstrates the variation of the community structure that explained by anti-desiccant in the first and second dimension, respectively

Anti-desiccant altered fungal community structure in ‘Vardar Valley’ but not ‘Justin Brouwers’ boxwood shoots. Specifically, permutational multivariate analysis of variance analysis (PERMANOVA) shows that anti-desiccants accounted for 41.7% (*P* = 0.0010) and 29.8% (*P* = 0.0040) of the total variation in fungal community structure in ‘Vardar Valley’ in June and October, two months after the first and second treatments, respectively (Fig. 5). Particularly, Vapor Gard and Wilt-Pruf were the main contributors driving the differences, evidenced by a clear separation of them and the nontreated controls in the first dimension of the Principal Coordinates Analysis (PCoA) ordinations (i.e., x-axis of the PCoA plot) (Fig. 5). Anti-desiccant impact on the fungal community structure of ‘Justin Brouwers’ was insignificant. Likewise, anti-desiccant impact on bacterial community structure was insignificant.

## Discussion

To our best knowledge, this is the first study evaluating the impact of anti-desiccant on the phyllosphere microorganisms at the whole community level. We discovered a wealth of fungal genera known as pathogens and saprophytes dominating the boxwood shoots. We also found that the predominant bacteria included many genera that have species and strains known to promote plant growth and health, agreeing with our previous study [10]. Foliar application of three film-forming anti-desiccants had more marked impact on fungi than bacteria in both community composition and structure, especially with Vapor Gard and Wilt-Pruf.

Diverse bacteria and fungi were identified from the two boxwood cultivars, extending our understanding of microbial assemblies in boxwood phyllosphere. Boxwood shoots were dominated by *Proteobacteria* at the phylum level and *Methylobacterium* at the genus level, consistent with our previous observation [10]. Many predominant bacterial genera, including *Methylobacterium*, *Stenotrophomonas*, *Bacillus*, *Sphingomonas*, and *Burkholderia*, have species and strains known to promote plant growth and health. For example, some *Methylobacterium* inoculants increased rice yield under the field condition [36] and *Burkholderia* SSG, an endophytic isolate of boxwood leaves promoted boxwood growth from 37.3 to 76.1% in 10 months [3]. These bacteria may play a pivotal role in the low-maintenance nature of boxwood. On the other hand, the dominant fungi were largely from the phylum *Ascomycota*, including *Shiraia*, *Alternariaster*, *Cladosporium*, *Ramularia*, and *Alternaria*. These fungi contain many species known as plant pathogens and saprophytes. For example, *Shiraia*-like fungi have been isolated from bamboo tissues as an endophytic parasite [37]. Detection of *Shiraia* as a dominant genus in boxwood is yet to be confirmed through isolation. Other predominant genera, such as *Alternaria* and *Cladosporium* were also isolated abundantly in other boxwood species and cultivars of the same species (i.e., *B. sempervirens*), particularly in the diseased boxwood plants [38–40]. Although these fungi do not directly cause any major boxwood disease, they may play a role in mediating plant defense reactions and accelerating plant senescence [41–44], making boxwood plants vulnerable to some pathogens [38, 40]. These results highlight the importance of understanding how phyllosphere fungal communities may have contributed to the Western North Carolina region as a hotspot for multiple boxwood diseases.

This study showed that the anti-desiccant treatments had differential impacts on the phyllosphere fungal and bacterial communities. Although the polymer ingredients are considered to be non-toxic to organisms [28], ANCOMBC showed that they impacted 96 fungal genera and 62 bacterial genera. Specifically, 54.2–67.6% of these fungi were suppressed by anti-desiccant treatments, while over 98.0% of the impacted bacteria were promoted. The mechanisms by which these polymer compounds interacted with these fungal and bacterial genera are unclear at this time. Polymers coating could alter fungal spore adhesion to plant surfaces and affect gemination of the spore [45], in addition to providing a physical barrier that prevents fungal direct contacts. Sutherland and Walters [46] also reported three anti-desiccants changed the morphology of the appressoria and haustoria in *Blumeria graminis*. Some anti-desiccants also suppressed powdery mildew [32, 46], grey mold [33], leaf rust [47], and *Guignardia* leaf blotch [20]. Compared with fungi, bacteria on the plant surface can produce biofilm, a matrix of extracellular polymeric substances to provide adequate protection and mitigate environmental stresses [48–50]. Importantly, the coating layer produced by the film-forming anti-desiccants increases water availability without limiting nutrients leached from plants [27], this may enhance bacterial survival and other activities on the plant surface or inside the tissue [51, 52]. In this study, Vapor Gard (96% di-1-p-menthene) and Wilt-Pruf (25% di-1-p-menthene) had more pronounced effect in altering fungal community composition and structure than TransFilm (8.2% polymeric terpenes and 9.1% oxidized polyethylene), this may be due to the polymer concentration is much lower in TransFilm than the other two products. What caused these differences among the three anti-desiccants is yet to be elucidated in future studies. Additionally, the least changes were observed in the fungal communities in August, four months after anti-desiccant treatments, compared to the broad impacts at two months after treatment, indicating the effect of anti-desiccant may have faded with time. This was not unexpected due to the polymer coating would be naturally degraded, indicated by the recommended application intervals.

## Conclusions

This study characterized the shoot bacterial and fungal communities of ‘Vardar Valley’ and ‘Justin Brouwers’ boxwood and assessed the effect of three film-forming anti-desiccants on these microbial communities. We found that the boxwood phyllosphere was a hub for a wealth of fungal genera that are known as pathogens and saprophytes, but the identified bacterial communities included many genera having species and strains known to benefit plant growth and health. The abundance of several predominant fungi varied more between the two cultivars than that of the bacterial communities, this could be attributed to different plant age and size, cultural practices and microclimate. Foliar application of three film-forming anti-desiccants altered both fungal and bacterial community compositions, but the effect was more marked and suppressive on fungi than bacteria. These results advanced our understanding of the associated microbiome for this landmark plant and the impacts of film-forming anti-desiccants on plant microbiomes. This study also provides a good example for examining other anti-desiccant products for their potential effects on crop microbiomes, enabling growers to utilize the full benefits of anti-desiccant action while minimizing their negative impact on the beneficial microbes.

## Methods

### Study sites and boxwood cultivars

This study was done in 2021 at two sites in Western North Carolina; and these two sites were about 35 km apart. A portion of 5-year old plantings of *Buxus sempervirens* ‘Vardar Valley’ on a commercial farm were used at site (1) Similarly, 3-year old *B. sempervirens* ‘Justin Brouwers’ were grown at site (2) At the time of the study, ‘Vardar Valley’ plants were about 60 cm in height and 40 to 50 cm in width, and ‘Justin Brouwers’ plants were about 20 cm in height and 12 to 18 cm in width. Both cultivars are slow-growing boxwood plants, at a rate about 2.5 to 7.6 cm annually. Plantings at both sites had not been treated with any anti-desiccants, insecticides, or fungicides before the study. At site 1, fertilizer was applied in early April and two herbicides Roundup® (Bayer AG, Leverkusen, Germany) and Goal® (Nutrichem Co., Ltd., Beijing, China) were sprayed at label rates in late May to manage weed. However, neither fertilizer nor herbicides were used at site 2. Daily temperature and precipitation records from April to October were retrieved from Iowa Environmental Mesonet (https://mesonet.agron.iastate.edu/, accessed on 10/5/2022) using the closest weather stations EKNN7 (network NC_COOP) and RAVN7 (network NC_DCP) for sites 1 and 2, respectively (Table S1).

### Anti-desiccants treatments and sample collection

Three anti-desiccant products TransFilm®, Vapor Gard®, and Wilt-Pruf ® were selected for this study (Table 1). TransFilm contains 8.2% polymeric terpenes and 9.1% oxidized polyethylene. Vapor Gard contains 96% Pinolene (di-1-p-menthene), a terpenic polymer. Wilt-Pruf contains 25% Pinolene. These polymers are classified as film-forming anti-desiccants, as they produce a thin, flexible, and transparent physical layer on the leaf surface. The film hinders the loss of water from the plant but permits diffusion of carbon dioxide [22]. According to the manufacturer, TransFilm also contains diethanolamine, a surfactant compound toxic to microorganisms with an EC_50_ value of 73 mg/L. The recommended application intervals are 3 to 4 months for TransFilm and Wilt-Pruf, and 3 to 6 months for Vapor Gard (Table 1). Anti-desiccants were freshly prepared following label instructions: 75 ml, 48 ml, and 150 ml of TransFilm, Vapor Gard, and Wilt-Pruf were respectively mixed with 925 ml, 952 ml, and 850 ml of water to prepare one liter of emulsified solution for each product.

**Table 1:** Emulsified anti-desiccant products included in this study

Product	Manufacturer	Active ingredient	Recommended application	
			Rate (%)	Interval (month)
TransFilm	PBI-Gordon	Polymeric terpenes (8.18%) Oxidized polyethylene (9.12%)	7.50	3–4
Vapor Gard	Miller	Di-1-p-menthene (96%)	4.76	3–6
Wilt-Pruf	Miller	Di-1-p-menthene (25%)	15.00	3–4

A randomized complete block design with four replicates was used to include one plant for each of the three anti-desiccant treatments and one nontreated control in a block. Plants were spaced about 90 to 120 cm apart. The first application of anti-desiccants to boxwood canopy was done on April 13,2021, and the second application was conducted on August 26, 2021, using a customized boom sprayer driven by 4-gallon CO_2_ compression tank for improved coverage on all sides of the boxwood shrubs.

Shoot samples were taken on June 16 and October 18, two months after the application. Another sampling took place on August 26, 4.5 months after the first application (or right before the second application). At the time of sampling, there was no sign of phytotoxicity on any boxwood plants caused by the anti-desiccant. Ten to twelve 5- to 8-cm long boxwood shoots without any disease symptom were randomly selected and taken at the top and middle sections of each plant using a hand pruner (FELCO, Seattle, WA, USA). The pruner was sterilized with 75% alcohol and wiped dry using fresh paper towels between plants. The shoots from each plant were collected into a new Ziploc® (Bay City, MI, USA) bag and then placed in a cooler containing ice packs. Samples were transported to the laboratory at Virginia Beach, VA the next day and stored at -20 °C.

### Sample processing, and DNA extraction

From the stem tip, five 5- to 8-cm long shoots were arbitrarily selected from each of the total 96 replicate samples. For each shoot, ‘Vardar Valley’ had an average of 20 leaves, and ‘Justin Brouwers’ had an average of 40 leaves. We used liquid nitrogen to homogenize the shoot samples. The extraction of genomic DNA followed Qiagen Plant Mini Kit (Hilden, Germany) with minor modifications. Specifically, approximately 200 mg ground tissue was further homogenized using a MP FastPrep™ 24 (MP Bio, Irvine, CA, USA) at the speed of 4/s for 1 min in a sterilized zirconium bead tube (500 um garnet and 6 mm zirconium, PFMM 500-100-25U, OPS Diagnostics, Lebanon, NJ, USA) prefilled with 400 µl of AP1 buffer. Four µl of RNase A was added and vortexed for 3 s. Nuclease free water was used to dissolve DNA from the MB Spin Column membrane. The DNA was stored at -20°C.

### PCR amplification

PCR amplification of full length 16 S rRNA amplicon followed the protocol in our previous study [10]. The extended ITS regions were amplified with the fungal-specific primers NSA3 and NLC2 [53]. DNA samples were first diluted to 10 ng/µl and a 50 µl PCR reaction mixture was prepared to include 1 µl of the primers, 5 µl of 10x Buffer, 4 µl of dNTP, 0.2 µl of the Takara® polymerase (Takara Bio, San Jose, CA, USA), and 1 µl of the template DNA. The PCR thermal conditions for amplifying the 16 S rRNA gene and ITS regions were the same and detailed in the previous study [10]. The Wizard® SV Gel and PCR clean-up system (Promega, WI, USA) was used to clean up the PCR products.

### Library preparation and nanopore MinION sequencing

Nanopore SQK-LSK110 ligation kit and EXP-PBC096 barcode kit (Oxford Nanopore Technology, Oxford, UK) were used to prepare multiplexed sequencing libraries. Procedures and thermal conditions followed the corresponding kit protocols. The AMPure XP beads (Beckman Coulter Life Science, Indianapolis, IN, USA) were used for each clean-up step. Sixteen of each barcoded 16 S rRNA and ITS amplicon libraries were pooled with equal molar amount of DNA to a total of 1 µg library for each sequencing run. Library loading followed the Nanopore priming and loading protocol.

### Base-calling, chimera removal, and taxonomy assignments

Nanopore live base-calling and chimera removal for 16 S rRNA reads were specified in Li et al. [10]. To removing chimeras in the ITS amplicon sequences, the full “UNITE + INSD” dataset (for fungi, version 8.3) [54] was used and curated [10] using the Quantitative Insights Into Microbial Ecology (QIIME2) [55] and the REference Sequence annotation and CuRatIon Pipeline (RESCRIPt) [56]. The length of the reference sequences was set at a minimum of 1,000 bp. Minimap2 [57] and yacrd [58] were implemented to pick chimera sequences following Cuscó et al. [59]. To assign fungal taxonomy, the chimera free sequences were then aligned to the curated “UNITE + INSD” reference database with Minimap2 and the top alignment was selected [59]. Here, we adopted the terminology ‘Operational Taxonomic Unit’ (OTU) to describe the taxonomy-assigned sequences. Downstream statistical analyses and visualization were carried out with R software (version 4.2.2) [60]. In R, the sequences were further pruned to remove samples with less than 1,000 sequences and OTUs less than 10 sequences across all the samples. Boxwood chloroplasts sequences were also removed from the 16 S rRNA sequences. Sample coverage was checked using the phyloseq_coverage function from the metagMisc package [61].

### Statistical analyses

#### Differential abundance analysis

Analysis of Compositional of Microbiomes with Bias Correction (ANCOM-BC, version 2.0.2) [62] was used with the ancombc2 function to identify differentially abundant bacterial and fungal genera as a function of anti-desiccant for each of the two cultivars and three sampling months. The maximum number of iterations was set to 500 for both iterative MLE (parameter: iter_control) and E-M (parameter: em_control) algorithms. The False Discovery Rate (FDR) [63] was used to correct *P* values from multiple tests at the significance level of 0.05.

### Microbial community structure

Microbial community structure was measured using the Bray-Curtis dissimilarity index [64] for each cultivar across three sampling months. OTU counts were first normalized with Hellinger transformation [65] by implementing the microbiome package (version 1.20.0) [66], and the dissimilarity distance matrix was then calculated using the vegdist function from the vegan package (version 2.6.4) [67]. Principal coordinate analysis (PCoA) was used to ordinate the dissimilarity matrix. Anti-desiccant effect on microbial community structure was tested using the permutational multivariate analysis of variance analysis (PERMANOVA) [67] with 1,000 permutations. The distances among the anti-desiccant treatments on the first and second dimensions were compared using Tukey’s honestly significant difference (HSD) test. Significance level of all tests was set at 0.05.

### Electronic supplementary material

Below is the link to the electronic supplementary material.

Supplementary Material 1

## Data Availability

The based-called and demultiplexed FASTQ reads are deposited at the National Center for Biotechnology Information Sequence Read Archive (NCBI SRA) under BioProject ID: PRJNA852261. R scripts used in this study are publicly available at https://github.com/lixiaopi1985/Anti-desiccant_impact.git.

## List of Abbreviations

ANCOM-BC: Analysis of compositional of microbiomes with bias correction
FDR: False discovery rate
ITS: Internal transcribed spacer
OTU: Operational taxonomy unit
PERMANOVA: Permutational multivariate analysis of variance analysis
PCoA: Principal coordinates analysis
PCR: Polymerase chain reaction
QIIME2: Quantitative insights into microbial ecology
rRNA: Ribosomal RNA
RESCRIPt: Reference sequence annotation and curation pipeline
