# Supplementary material for: Boxwood phyllosphere fungal and bacterial communities and their differential responses to film-forming anti-desiccants
Source: BMC Microbiol. 2023 Aug 12;23:219. doi: 10.1186/s12866-023-02956-0 (PMC10422719; doi:10.1186/s12866-023-02956-0)
Supplement: Supplementary file 1 — Supplementary Material 1 [file 12866_2023_2956_MOESM1_ESM.docx]

Supplemental tables

Table S1. Weather conditions for sites 1 and 2 during each sampling period

|  | Average daily temperature (°C) | | |  | Total precipitation (mm) | | |
| --- | --- | --- | --- | --- | --- | --- | --- |
|  | 4/1 – 6/16 | 6/17 – 8/26 | 8/27 – 10/18 |  | 4/1 – 6/16 | 6/17 – 8/26 | 8/27 – 10/18 |
| Site 1 | 16.7±5.44 | 23.8±1.97 | 20.4±3.13 |  | 191 | 334 | 149 |
| Site 2 | 17.0±5.21 | 23.8±2.23 | 20.1±3.11 |  | 229 | 421 | 124 |

Supplemental figures


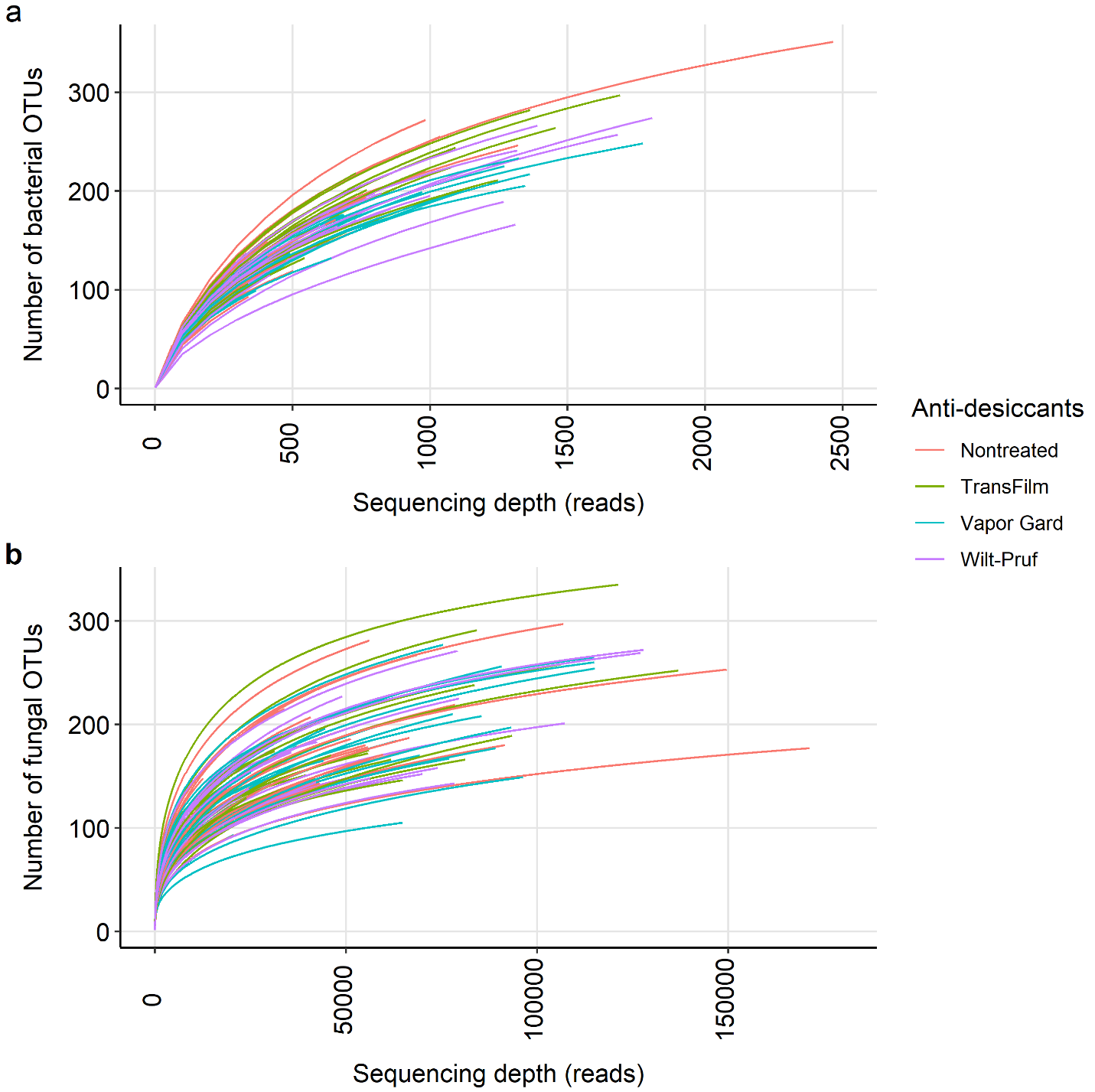


Figure S1. Rarefaction curves of the clean 16S rRNA (**a**) and ITS (**b**) reads by antidesiccant. The rarefaction curve was drawn with the ampvis2 package with 100 step-size.


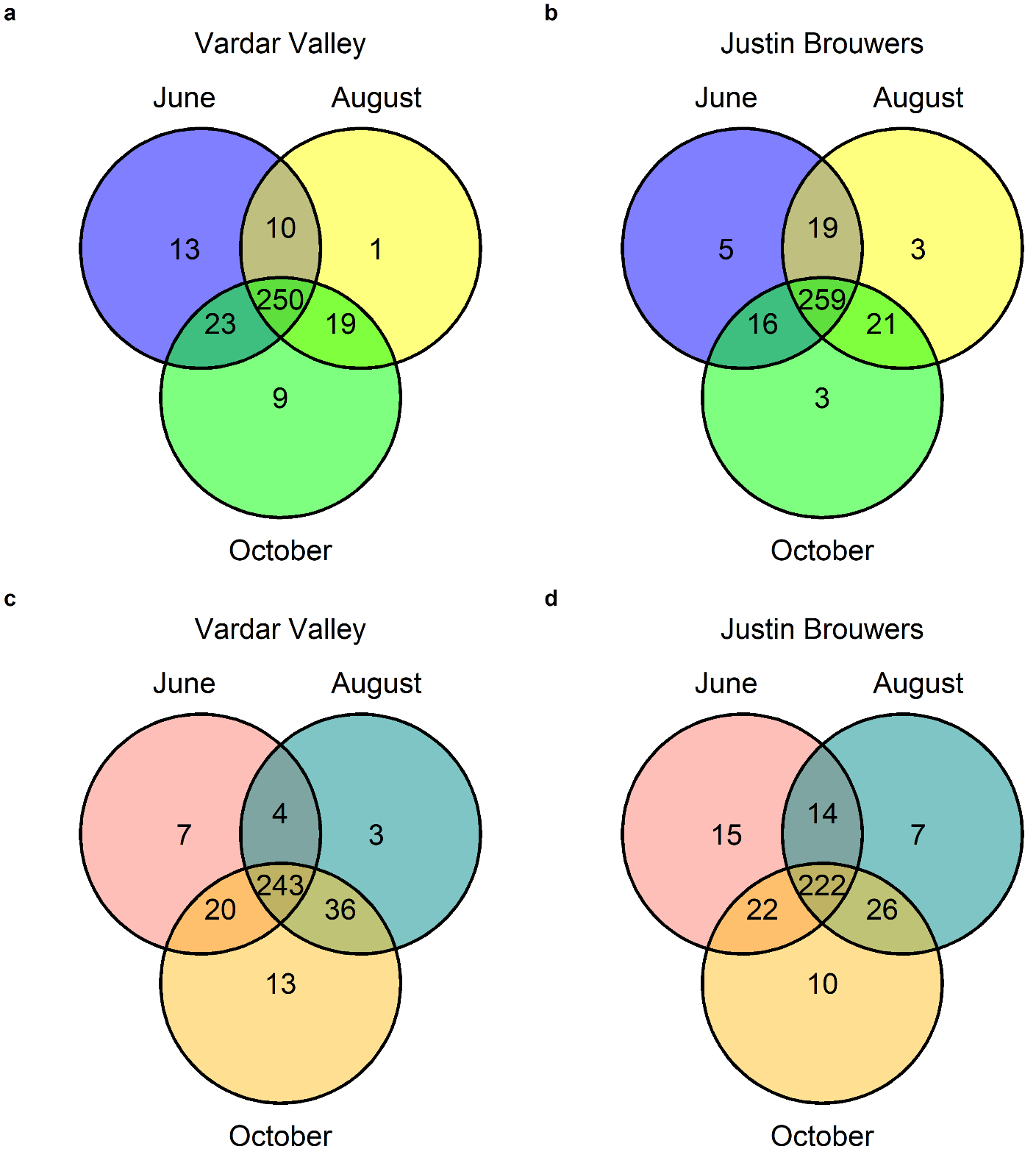


Figure S2. Bacterial (**a** and **b**) and fungal (**c** and **d**) genera identified from shoots of ‘Vardar Valley’ boxwood and ‘Justin Brouwers that are unique to or shared among the three sampling months - June, August, and October.
